# Short-Term Test-Retest Reliability of Electrically Evoked Cortical Auditory Potentials in Adult Cochlear Implant Recipients

**DOI:** 10.3389/fneur.2020.00305

**Published:** 2020-04-28

**Authors:** Meghan Pike, Leigh Biagio-de Jager, Talita le Roux, Louis M. Hofmeyr

**Affiliations:** Department of Speech Language Pathology and Audiology, University of Pretoria, Pretoria, South Africa

**Keywords:** cochlear implant, test-retest reliability, cortical auditory evoked potentials, late latency auditory evoked potentials, electrical evoked responses, aided

## Abstract

**Background:** Late latency auditory evoked potentials (LLAEPs) provide objective evidence of an individual's central auditory processing abilities. Electrically evoked cortical auditory evoked potentials (eCAEPs) are a type of LLAEP that provides an objective measure of aided speech perception and auditory processing abilities in cochlear implant (CI) recipients.

**Aim:** To determine the short-term test-retest reliability of eCAEPs in adult CI recipients.

**Design:** An explorative, within-subject repeated measures research design was employed.

**Study Sample:** The study sample included 12 post-lingually deafened, unilaterally implanted adult CI recipients with at least 9 months of CI experience.

**Method:** eCAEPs representing basal, medial and apical cochlear regions were recorded in the implanted ears of each participant. Measurements were repeated 7 days after the initial assessment.

**Results:** No significant differences between either median latencies or amplitudes at test and retest sessions (*p* > 0.05) were found when results for apical, medial and basal electrodes were averaged together. Mean intraclass correlation coefficient (ICC) scores averaged across basal, medial and apical cochlear stimulus regions indicated that both consistency and agreement were statistically significant and ranged from moderate to good (ICC = 0.58–0.86, *p* < 0.05). ICC confidence intervals did demonstrate considerable individual variability in both latency and amplitudes.

**Conclusion:** eCAEP latencies and amplitudes demonstrated moderate to good short-term test-retest reliability. However, confidence intervals indicated individual variability in measurement consistency which is likely linked to attention and listening effort required from the CI recipients.

## Introduction

A cochlear implant (CI) recipient's speech perception and auditory processing abilities with a CI are strongly linked to the integrity of that individual's central auditory pathways (1). Late latency auditory evoked potentials (LLAEPs) are used to determine the neurophysiological changes that occur in the cortical regions of the auditory pathway with respect to specific skills that include attention, auditory discrimination, integration and memory (2). LLAEPs therefore provide objective evidence of an individuals' central auditory processing abilities (3). Cortical auditory evoked potentials (CAEPs) are a type of LLAEP that is receiving increasing attention in literature for the purpose of providing an objective measure of aided speech perception and auditory processing abilities in CI recipients (3, 4).

CAEPs are voltage potentials which originate from various auditory structures in the brain in response to sound (5). These areas include the primary auditory cortex and the thalamic and auditory association areas (5). The characteristics of a CAEP response include a series of positive and negative peaks, which are known as the P1-N1-P2 complex (6). P1, a positive peak at 50 ms, which mainly arises from the primary auditory cortex, whereas N1 is seen as a negative deflection occurring at 100 ms with primarily frontocentral contribution (7). P2 has multiple generators in Heschl's gyrus and is seen as a positive peak at 175 ms (7). CAEP latency values indicate the neural travel time in response to auditory stimulation, with P1 latency, in particular, reflecting accumulated sum of delays in synaptic propagation through the peripheral and central auditory pathways (8, 9). P1 latency can be used to infer the development of auditory pathways in children fitted with CI and speech recognition outcomes (10, 11).

In addition to threshold estimation, CAEPs are used in the clinical setting to provide an estimate of an individual's supra-threshold processing abilities (12) as well as to examine plasticity-related changes that occur in the brain (13). Aided CAEPs can be defined as an auditory evoked potential response that is elicited from a hearing aid (HA) user or CI recipient using stimuli that are processed by the individual's HA or CI (14). The main purposes of recording aided CAEPs in HA users is to verify that the amplified signal created by the HA is being successfully processed by the brain and to examine any changes that occur in the brain as a result of plasticity (13, 14).

Aided CAEPs have also been used to assist with validation and verification of HA fittings and to examine any changes that occur in the brain as a result of plasticity (13). An early study compared CAEPs and aided CAEPs in young children (<2 years of age) with severe to profound sensorineural hearing loss who were fitted with conventional HAs (15). Responses were elicited through click and tonal stimuli and indicated that aided CAEPs were at least 20dB nHL better when compared to unaided results (15). Glista et al. (16) compared the aided CAEP response in HA users and age matched normal hearing individuals. Results indicated that aided CAEPs were larger in amplitude when recorded in HA users compared to normal hearing individuals. Furthermore, there was a strong association between the presence of repeatable aided CAEPs in HA users and the degree of audibility (16). The presence of repeatable aided CAEP responses at a suprathreshold intensity in HA users therefore provides physiological evidence that the stimuli presented is being detected up to the level of the auditory cortex (16). Additionally, interrater reliability analysis using the Kappa statistic to examine consistency of waveform analysis between two raters was also conducted which was found to be perfect [ = 1; (16)]. Similarly, with unaided CAEP, Angel (17) reported good interrater correlation.

In order to record CAEPs in CI recipients, aided CAEPs or electrically evoked cortical auditory evoked potentials (eCAEPs) can be measured. When measuring eCAEPs, the stimulus bypasses the speech processor and is directly transmitted to the implanted device, therefore eradicating any pre-processing effects created by the CI (18, 19). For CI recipients specifically, aided CAEPs have been used to not only assess auditory functioning and record developmental changes that occur post-implantation but also to assist in device programming (10, 20, 21). The aided CAEP response is modified by the CI settings and can therefore be used to determine the effects of various signal processing strategies on evoked neural activity (13).

Groenen et al. (10) compared aided CAEP latencies between adult CI recipients whose aided speech perception performance 2 years post-implantation was rated to be either “moderate” or “good” based on several speech perception tests. Results demonstrated that adult CI recipients with “good” speech perception outcomes obtained aided CAEP latencies and amplitudes that correlated with age-matched, normal hearing adults (10). However, adult CI recipients with “moderate” speech perception outcomes presented with reduced P2 amplitudes compared to CI recipients with “good” speech perception outcomes. The study suggested that the reduced P2 amplitude in users with “moderate” and speech perception provided evidence that the cochleotopical organization of the auditory cortex was less distinct than in these CI recipients with “good” speech perception outcomes (10).

Kelly et al. (3) also found similar latencies and amplitudes for CAEPs with well-defined morphology in normal hearing adults and aided CAEPs in post-lingually deafened adult CI recipients with at least 1 year of CI experience. However, results indicated that those CI recipients who were good performers, presented with a decreased P1 amplitude and an increased N1 amplitude when compared to poorer performing CI recipients (3). Aided CAEPs may consequently be used to predict an individual's performance with a CI (10) as speech perception outcomes correlate with aided CAEP amplitudes in adult CI recipients (3, 10, 13).

Aided CAEPs have been found to be reliably recorded in the sound field for individuals with and without a digital HA (6, 14, 22) as well as in CI recipients (18). Tremblay et al. (6) determined the short term test-retest reliability of a specific CAEP, namely the acoustic change complex, in seven normal hearing individuals between 23 to 31 years of age using naturally produced speech stimuli. The intra-class correlation (ICC) statistic of the grand mean responses indicated high short term test-retest reliability when CAEPs were recorded from the same individual [(6); ICC = moderate to good across individuals]. The study concluded that changes in the morphology of the CAEP response noted over a short period of time are therefore likely to reflect changes in neural activation in response to speech.

Czarniak (18) determined the test-retest reliability of aided CAEPs in CI recipients and found aided CAEPs, through the use of speech stimuli, to be repeatable across test sessions. The test-retest reliability was determined through a repeated measures analysis and various scatter plots (18). However, to the author's knowledge, there is no published literature addressing the test-retest reliability of eCAEPs in CI recipients specifically. Further understanding of eCAEPs, in comparison to aided CAEP results, may offer a better understanding with regards to the variability that exists in CI recipients' performance (18).

The increased need to utilize objective measures, such as eCAEPs in CIs can be linked to the fact that children are being implanted at earlier ages and require more objective programming options (19). Furthermore, these objective measures can provide important information that will add to the understanding of the variability of CI outcomes (19). Although the validity of aided CAEPs in the clinical setting has been measured, there is a lack of published literature specifically addressing the test-retest reliability of eCAEPs in CI recipients. It is important to determine the test-retest reliability of eCAEPs in CI recipients in order to appraise the consistency of the measurements and to draw realistic conclusions based hereon (23). The present study therefore aimed to determine the short-term test-retest reliability of eCAEPs in CI recipients.

## Methods

An explorative, within-subject repeated measures research design was employed. Institutional ethics committee approval was obtained prior to the commencement of data collection (GW20170307HS).

### Participants

Twelve post-lingually deafened, adult (>18 years) CI recipients (five males, seven females) aged 27–67 years (mean = 50.3 years, *SD* = 12.9) participated in the study and were recruited from two CI programs in South Africa, namely the Pretoria Cochlear Implant Unit and the Johannesburg Cochlear Implant Centre. A sample size calculation was performed with P_o_ (the minimum expected acceptance reliability) of 40% (24) and P_1_ (the expected reliability) of 70%, power (1-β) of 0.800, and significance level of 0.050. A recommended sample size of 13 participants was calculated (25). One participant did not return for retest, and the sample was reduced to 12 participants.

Informed consent was obtained from all participating CI recipients prior to data collection. All participants were unilaterally implanted with a Nucleus CI24RE (CA) or a CI512 device from Cochlear©, using either a CP810, CP910, or CP920 speech processor. Each participant presented with at least a severe sensorineural hearing loss in the non-implanted ear. A severe sensorineural hearing loss was defined as a pure tone average of 71-90 dB HL (26). Documented etiological/risk factors of severe to profound hearing loss included inner ear autoimmune condition (*n* = 1), Ushers syndrome (*n* = 1), Waardenburg syndrome (*n* = 1), Rubella (*n* = 1), repeated otological surgical procedures (*n* = 1), and ototoxic medication (*n* = 3). For all participants, surgery was uneventful and a full electrode insertion was achieved. Duration of deafness prior to CI use ranged from 1.1 to 45.8 years (mean = 20.1 years, *SD* = 18). At the time of data collection all participants had CI experience of at least 9 months and had at least 20 active electrodes. All participants were oral communicators with the required receptive language abilities to understand the instructions given for testing and were able to ask for clarification if necessary.

### Equipment and Procedure for Data Collection

eCAEPs were recorded using the Interacoustics Eclipse EP25 Auditory Evoked Response System V1.3 software (Interacoustic A/S, Assens Denmark), calibrated in accordance with ISO 389-1 (2018). Calibration of the Interacoustics Eclipse EP25 Auditory Evoked Response System V1.3 software (Interacoustic A/S, Assens Denmark) included peak equivalent sound pressure level (peSPL) and normal hearing level (nHL) calibration. With regards to peSPL dB value, the maximum acoustic level was calibrated to match the dBSPL level of continuous tones obtained on a sound level meter. A correction factor was used to compensate for the difference in perceived loudness of very brief stimuli like clicks and tone bursts. Longer duration tone bursts as are used for LLAEPs make use of peak-to-peak equivalent reference equivalent threshold sound pressure levels values as described in ISO 389-1 (27). The Custom Sound EP 5.0 (Cochlear©) software was used to stimulate participants' CI devices during the recording of the eCAEPs.

### Protocol and Parameters Used to Measure eCAEPs

An electrical stimulus was presented at a suprathreshold current level that was comfortable for each CI recipient, via the Custom Sound EP 5.0 (Cochlear©) software. Stimulation and repetition rate were presented at 900 and 0.9 Hz, respectively with a pulse width of 25 μs and a 7 μs inter phase gap. A total of 450 pulses per burst were utilized with three sweeps of 20–40 averaged stimuli for both test and retest sessions.

### Procedure

The sites of electrode placement were cleaned with Nuprep abrasive paste. Each silver chloride cup electrode was filled with Ten20 Neurodiagnostic electrode paste. In order to record the eCAEP response, the inverting electrode was placed on the contralateral mastoid (Mc-inverting electrode) in order to minimize CI stimulus artifacts (3). The non-inverting was placed on Cz with ground on Fpz. Impedances were required to be below 3 kΩ prior to commencement of testing (3). Each participant was seated on a slightly reclined, comfortable chair in a sound treated room.

Whilst recording the eCAEP response, the electrical stimulus was presented through the CI, via the Custom Sound EP 5.0 (Cochlear©) software allowing the speech processor to be bypassed and the direct stimulation of the CI to be controlled (4). This software was linked, via a trigger cable, to the Interacoustics Eclipse EP25 Auditory Evoked Response System V1.3 software (Interacoustic A/S, Assens Denmark). The electrical stimulus was set at a comfortable current level and it was ensured that each participant was kept mentally alert, with eyes open but downcast during the test procedure. eCAEPs were measured on three different electrodes along the electrode array, representing apical, medial, and basal cochlear regions. Once a comfortable current level was obtained for each cochlear region, these current levels were noted and the same stimulus levels selected for the retest session.

A minimum of three waveforms were averaged for each eCAEP response from each session. Two independent and experienced evaluators evaluated the waveforms. Both evaluators were required to be in agreement with regards to the analysis of the amplitude and latencies of the waveforms measured.

The eCAEP latencies were defined as the time in millisecond from stimulus onset to peak amplitude value (18, 28). The N1 and P2 amplitudes were measured from the baseline-to-trough (29) and baseline-to-peak of the N1 and P2 response, respectively (18), whilst the N1-P2 response was measured from trough to peak (30).

The aforementioned procedure was then repeated 7 days later and results were compared to the initially obtained results, in order to determine the short-term test-retest reliability of aided CAEPs in adult CI recipients. All CI settings remained unchanged at the retest session and the exact same testing protocol and stimulus parameters were utilized with regards to the recording of the aided CAEP response.

### Analysis

Descriptive and inferential statistics were used in order to compare the latencies (msec) and amplitudes (μV) of the aided eCAEP waves between sessions.

In order to evaluate the normality of distribution of latency and amplitude measures, the Shapiro-Wilk test, with histograms and normal Q-Q plots was used. The Shapiro-Wilk test was not significant (*W* = 0.863–0.973; *p* > 0.05) for 30 out of the 40 eCAEP variables. Due to the ten variables which were not normally distributed (*W*= 0.664–0.855; *p* < 0.05), by visual inspection of the histograms and normal Q-Q plots, and due to the small sample size (viz. 12 participants), non-parametric, distribution free statistics were used, namely the Wilcoxon signed rank test, to compare the median difference between paired observations, namely for the latencies and amplitudes of the eCAEPs at test and retest. The ICC was employed to determine the reliability of the results and to reflect both the degree of correlation, and the degree of agreement between measures. It is recommended that the reliability of a measure not only be evaluated by looking at difference at test and retest, as was achieved using Wilcoxon signed rank test, but also in terms of both consistency and agreement (23, 31). It was for this reason that the ICC measures of both consistency and agreement were determined. ICC estimates and their 95% confident intervals were therefore calculated based on a averaged measures, consistency, 2-way mixed-effects model, as well as averaged measures, agreement, 2-way mixed-effects model (23). The variables that were not normally distributed were strongly positively skewed. A logarithmic transformation (log_10_) of all of the data was therefore conducted (32) and data was normally distributed thereafter as confirmed by Shapiro-Wilk (*W* = 0.870–0.981; *p* > 0.05). ICC co-efficients ranged from 0 for dissimilar latencies and amplitudes, to 1 for identical latencies and amplitudes. ICC estimate values <0.5, between 0.5 and 0.75, between 0.75 and 0.9, and more than 0.90 indicated poor, moderate, good, and excellent reliability, respectively, based on the 95% confident interval (23). Statistics were calculated using SPSS statistical package version 24 (SPSS Inc., Chicago, IL). For ICC measures of agreement and consistency, and for Wilcoxon signed rank test, a significance level of 0.05 was adopted.

## Results

**Figure 1:**
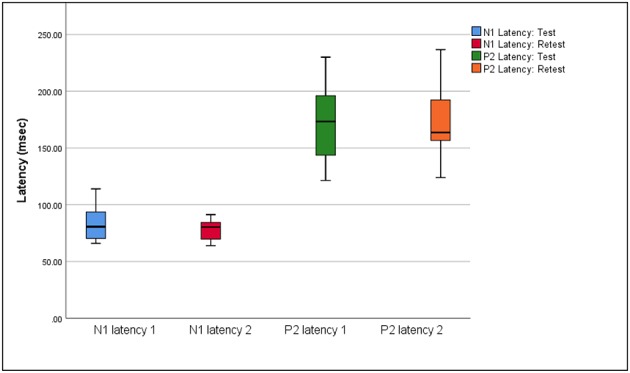
Median, interquartile range, minimum, and maximum N1 and P2 latencies at test and retest (*n* = 12).

**Figure 2:**
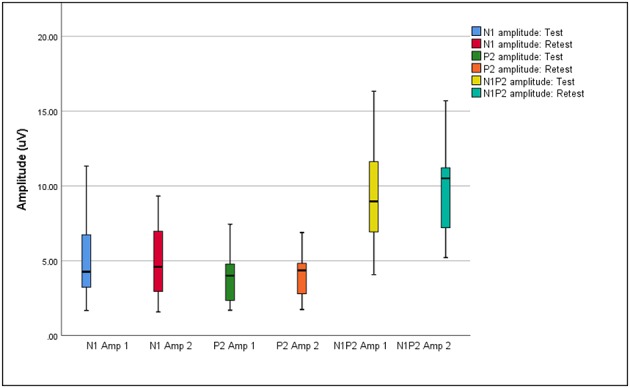
Median, interquartile range, minimum, and maximum N1, P2, and N1P2 amplitudes at test and retest (*n* = 12).

Box plots of median eCAEP latencies and amplitudes (representing basal, medial and apical cochlear regions) at test and retest are presented in Figures 1, 2, respectively. Individual eCAEP latencies and amplitudes at test and retest are presented in Data Sheet 1.

The median N1 latency ranged from 71.00 to 84.00 ms and the P2 latency ranged from 163.00 to 178.00 when measured at basal, medial and apical electrodes. Median amplitudes ranged from 3.04 to 5.07 μV for the N1 amplitude, 2.62 to 4.67 μV for the P2 amplitude, and 7.31 to 10.41 μV for the N1-P2 amplitude. Wilcoxon signed-rank test indicated no significant differences between median latencies and amplitudes (representing basal, medial, and apical electrodes/cochlear regions) between the test and retest sessions (*p* > 0.05), except for the P2 amplitude on the apical electrode (*z* = 2.045, *p* = 0.041). When results for apical, medial, and basal electrodes were averaged together, no significant differences between either median latencies or amplitudes between test and retest sessions (*p* > 0.05) were found.

eCAEP intra-participant test-retest ICC reflecting agreement and consistency with 95% confidence intervals (*n* = 12) are reflected in Table 1.

**Table 1:** eCAEP intra-participant test-retest ICC reflecting agreement and consistency with 95% confidence intervals (*n* = 12).

		**Electrode**	**Consistency**			**Agreement**		
			**ICC**	**Sig**.	**95% confidence interval**	**ICC**	**Sig**.	**95% confidence interval**
Latency	N1	Basal	0.54	0.101	−0.57 to 0.87	0.56	0.101	−0.55 to 0.87
		Medial	0.53	0.116	−0.65 to 0.86	0.54	0.116	−0.73 to 0.87
		Apical	0.72^*^	0.023	0.038–0.92	0.70^*^	0.023	0.06–0.91
		Mean	0.76^*^	0.013	0.17–0.93	0.76^*^	0.013	0.20–0.93
	P2	Basal	0.84^**^	0.003	0.45–0.95	0.84^**^	0.003	0.47–0.95
		Medial	0.32	0.265	−1.36 to 0.81	0.34	0.270	−1.69 to 0.82
		Apical	0.75^*^	0.015	0.13–0.93	0.76^*^	0.015	0.16–0.93
		Mean	0.78^**^	0.009	0.24–0.94	0.80^**^	0.009	0.25–0.94
Amplitude	N1 baseline-to-trough	Basal	0.55	0.100	−0.56 to 0.87	0.55	0.100	-0.52 to 0.87
		Medial	0.59	0.077	−0.42 to 0.88	0.60	0.077	−0.44 to 0.89
		Apical	0.93^***^	0.000	0.76–0.99	0.98^***^	0.000	0.72–0.98
		Mean	0.85^**^	0.002	0.48–0.96	0.86^**^	0.002	0.50–0.96
	P2 baseline-to-peak	Basal	0.62	0.060	−0.31 to 0.89	0.63	0.060	−0.28 to 0.89
		Medial	−0.23	0.632	−3.28 to 0.65	−0.24	0.632	−3.84 to 0.65
		Apical	−0.50	0.743	−4.21 to 0.57	−0.37	0.743	−2.19 to 0.54
		Mean	0.58	0.086	−0.48 to 0.88	0.58	0.086	−0.44 to 0.88
	N1-P2	Basal	0.86^**^	0.001	0.53–0.96	0.87^**^	0.001	0.55–0.96
		Medial	0.41	0.201	−1.07 to 0.83	0.42	0.201	−1.16 to 0.84
		Apical	0.79^**^	0.007	0.29–0.94	0.76^**^	0.007	0.20–0.93
		Mean	0.74^*^	0.019	0.78–0.92	0.74^*^	0.0019^*^	0.11–0.92

*Sig, significance*.

* *Significant (p < 0.05)*.

** *Significant (p < 0.01)*.

*** *Highly significant (p < 0.001)*.

Excellent consistency and agreement (ICC > 0.9) was seen for the N1 amplitude (apical cochlear regions). Good consistency and agreement (ICC = 0.75–0.9) was obtained for the P2 latency (basal and apical cochlear regions), as well as the N1-P2 amplitude (basal and apical cochlear regions). Moderate consistency and agreement (ICC = 0.5–0.75) was obtained for the N1 latency (across all cochlear regions), the N1 amplitude (basal and medial cochlear regions), as well as the P2 amplitude (basal cochlear region). Poor consistency and agreement (ICC < 0.5) was obtained for P2 latency (medial cochlear region), the P2 amplitude (medial and apical cochlear regions), as well as the N1-P2 amplitude (medial cochlear region). The ICC confidence intervals indicated very broad measures of consistency and agreement ranging from poor to excellent. Mean ICC scores averaged across basal, medial, and apical cochlear stimulus regions indicated that both consistency and agreement ranged from moderate to good (ICC = 0.58–0.86).

Statistically significant ICC (*p* < 0.05) were evident between test and retest for all amplitudes and latencies, except for N1 latency (basal and medial cochlear regions), P2 latency (medial cochlear region), N1 amplitude (basal and medial cochlear regions), P2 amplitude (across all cochlear regions), and N1-P2 amplitude (medial cochlear regions). ICC values were highly significant for the N1 amplitude measured with apical electrode stimulation. Mean ICC scores averaged across stimulus regions indicated statistically significant (*p* < 0.05) ICC values between test and retest for all eCAEP amplitudes and latencies except the P2 amplitude.

## Discussion

Short-term test-retest reliability of eCAEPs was determined in 12 adult CI recipients. eCAEPs were performed on 12 post-lingually deafened, unilaterally implanted adult CI recipients and repeated 7 days later. No significant median difference (*p* > 0.05) was measured between test and retest of N1 and P2 latencies and amplitudes, when averaged across electrodes. Statistically significant moderate to good ICC scores of consistency and agreement were measured for averaged N1 and P2 latencies and amplitudes averaged across basal, medial and apical electrode stimulation sites.

eCAEP latencies and amplitudes were also reported separately as measured from basal, medial and apical electrodes. The P2 amplitude as measured on the apical electrode was the only variable which was significantly different at test and retest (*p* = 0.041). Averaged across the three electrodes, no significant median difference was found between test and retest for N1 and P2 latencies and amplitudes (*p* > 0.05). The median N1 (71.00–84.00 ms) and P2 (163.00–178.00 ms) latencies of the eCAEP response measured at basal, medial and apical electrodes at both test and retest fell within the standard range for normal hearing adults [i.e., 75–150 ms for N1 latency; 150–250 ms for P2 latency; (33)]. These results indicated that central auditory processing up to the level of the auditory cortex was relatively intact in the current sample of post-lingually deafened adult CI recipients (3, 33, 34). eCAEP latencies measured in the current study were also in agreement with aided N1 and P2 latencies obtained for CI recipients in previous studies (10, 35).

All P1 and N2 amplitudes and latencies showed moderate to good test-retest reliability when averaged across electrodes (ICC = 0.58–0.86 ms). The present study's findings are consistent with previous literature on the reliability of CAEPs in response to tones in normal hearing individuals (36, 37). A number of studies also confirmed that aided CAEPs can be reliably recorded in the sound field for individuals with and without a digital HA (6, 14, 22). P1 and N2 amplitude and latency ICC scores averaged across electrodes ranged from moderate to good and therefore supports the findings of Tremblay et al. (6) who obtained ICC scores ranging from moderate to good consistency and agreement when recording a specific type of CAEP, namely the acoustic change complex, over short intervals in normal hearing individuals.

Although exceptions do occur, most CI recipients present with superior attention skills when compared to profoundly deafened individuals who are fitted with HAs or vibrotactile devices (38). Nevertheless, CI recipients are still found to present with poorer attentional abilities when compared to normal hearing individuals (38, 39). CI recipients require increased listening effort when compared to normal hearing individuals and more so for unilaterally implanted recipients as opposed to bilaterally implanted individuals (40). This increased expenditure of effort on listening can result in lower levels of attention (41). Thus, the effects of attention on cognitive processing could influence the recording of eCAEPs (42). This was evident when examining the confidence intervals obtained in this study, particularly with regards to the P2 amplitude and the N1 and P2 latencies. Wide confidence intervals may have been linked to a variety of factors, including the broad range of age, and duration of deafness, as well as the small sample size. In addition, endogenous auditory evoked responses such as CAEP are known to be highly dependent on the stimulus context, such as subject state, attention to the stimulus and cognition, or the task required of the participant (43).

In the current study, the removal of residual noise was attempted by placing the reference electrode on the contralateral mastoid, through signal and trace averaging, and through the use of an artifact rejection algorithm. However, residual noise levels at test and retest were not quantified. It is therefore possible that residual noise levels may not have been comparable between sessions and may have played a role in the variability of results obtained in this study (44). It is recommended that future studies that measure test-retest reliability for eCAEPs control for residual noise levels between test sessions (45).

Limitations of the current study include the limited sample size, one less than is recommended by the sample size calculation. In an effort to ameliorate this, test-retest reliability was repeatedly measured on three points along the electrode array for each participant. The test-retest ICC's reported and the use of averaged measurements to determine ICC, as was done in the current research, is influenced by the inter-rater reliability and as such, needs to be further explored. Previous evaluation of interrater reliability of aided CAEPs in children and adults indicated perfect to good agreement between two raters (16, 17).

## Conclusion

eCAEP latencies and amplitudes demonstrated moderate to good short-term test-retest reliability. Therefore, eCAEPs can be utilized in the clinical setting for adult CI recipients to monitor variations in the neural detection of time-varying cues over time. Given the reliability of eCAEPs, these auditory evoked potentials can be applied to study and monitor neural processing in adult CI recipients.

## Data Availability Statement

The dataset generated for this study is available on the University of Pretoria Research Data Repository (doi: 10.25403/UPresearchdata.11819589). Please note that the dataset is confidential and requests to access the dataset will be considered on a case by case basis. Requests for access should be sent to Leigh Biagio-de Jager (email: leigh.biagio@up.ac.za).

## Ethics Statement

The studies involving human participants were reviewed and approved by University of Pretoria. The patients/participants provided their written informed consent to participate in this study.

## Author Contributions

MP: investigation (data collection), data curation, writing of original draft and review, and project administration. LB: conceptualization, methodology, validation, formal analysis, review, and supervision. TR: conceptualization, methodology, validation, review, and supervision. LH: validation, review, and supervision.

## Conflict of Interest

The authors declare that the research was conducted in the absence of any commercial or financial relationships that could be construed as a potential conflict of interest.

## Abbreviations

CAEPs: cortical auditory evoked potentials
eCAEPs: electrically evoked cortical auditory evoked potentials
CI: cochlear implant
ICC: intraclass correlation coefficient
LLAEP: late latency auditory evoked potentials.
