# Supplementary material for: Short-Term Test-Retest Reliability of Electrically Evoked Cortical Auditory Potentials in Adult Cochlear Implant Recipients
Source: Front Neurol. 2020 Apr 28;11:305. doi: 10.3389/fneur.2020.00305 (PMC7198904; doi:10.3389/fneur.2020.00305)
Supplement: Supplementary file 1 [file Data_Sheet_1.pdf]

Appendix A

Individual participant eCAEP latencies and amplitudes at test and retest (n=12)

		Electrode	1 Test	1 Retest	2 Test	2 Retest	3 Test	3 Retest	4 Test	4 Retest	5 Test	5 Retest	6 Test	6 Retest	7 Test	7 Retest	8 Test	8 Retest	9 Test	9 Retest	10 Test	10 Retest	11 Test	11 Retest	12 Test	12 Retest
Latency (msec)	N1	Basal	158	92	72	68	82	68	68	66	64	66	98	86	72	68	108	90	68	84	92	72	70	74	66	92
		Medial	98	84	74	74	86	90	66	64	64	66	106	104	68	70	86	80	82	78	98	98	96	84	60	90
		Apical	86	98	64	70	102	92	64	68	64	66	92	66	72	68	98	80	98	84	68	66	70	70	92	92
		Mean	114	91,33	70	70.67	90	83,33	66	66	64	66	98,67	85.33	70,67	68,67	97,33	83,33	82.66	82	86	78,67	78,67	76	72.67	91,33
	P2	Basal	260	236	154	224	194	180	98	98	144	160	172	166	98	154	184	160	122	154	178	156	168	170	206	216
		Medial	138	240	136	156	154	208	130	116	136	142	190	198	182	174	242	166	150	150	296	156	190	190	186	222
		Apical	268	260	204	232	198	154	136	158	140	160	220	146	162	124	168	170	144	182	216	166	124	122	188	196
		Mean	222	245,33	164,67	204	182	180,67	121,33	124	140	154	194	170	147,33	150,67	198	165,33	138,67	162	230	159,33	160,67	160,67	193,33	211,33
Amplitude (µV)	N1 baseline -to- trough	Basal	0.23	4	5,25	6,69	3,23	2,4	9.7	10	9,46	6,11	2,45	5,26	10,2	13	2,85	3,32	1,77	1,02	1,61	6,08	5,1	1,45	2,58	3,44
		Medial	2,87	5,93	5,32	6,34	3,13	3,88	11.2	8.4	11,8	11,4	3,58	6,45	6,05	2,76	2,79	2,68	1,9	3,21	4,82	4,93	6,06	2,47	5,39	0,85
		Apical	2,29	2,06	8,01	7,83	3,89	4,42	13,1	9,6	8,69	6,11	4,88	3,92	5,61	4,35	3,52	3,63	1,39	0,53	5,98	10	3,74	1,22	5,26	3,84
		Mean	1,8	4	6,19	6,95	3,42	3,57	11.33	9.33	9,98	7,87	3,64	5,21	7,29	6,7	3,05	9,63	1,69	1,59	4,14	7	4,97	1,71	13,23	2,71
	P2 baseline -to-peak	Basal	4,2	0.57	3,58	3,97	4,46	2,15	0,7	0,48	0,37	3,83	2,6	2,29	9,74	5	2,32	1,56	9	4,92	5,54	2,62	8,84	6.45	4,19	2,63
		Medial	0,76	5,96	3,41	3,07	2,77	1,87	2,76	2,29	2,59	1,89	1,31	8,9	2,52	3,94	5,22	2,12	4,4	2,89	4,96	3,18	11,88	8,93	2,62	8,85
		Apical	1,86	7,86	5,11	5,26	4,72	1,23	2,34	4,2	2,14	3,83	3,42	3,42	1,22	4,93	4,61	5,56	1,83	3,05	7,2	8,22	1,62	5.29	3,28	9
		Mean	2,27	4,8	4,03	4,1	3,98	1,75	1,93	2,32	1,7	9,55	2,44	4,87	4,49	4,62	4,05	3,08	5,08	3,62	5,9	4,67	7,44	6.89	3,36	6.83
	N1-P2	Basal	4,44	4,57	8,83	10,66	7,7	4,56	13,47	13,03	9,09	9,95	5,07	7,55	20,01	18,05	5,17	4,88	10,77	5,94	7,15	3,46	13,94	17,91	6,77	7,07
		Medial	3,64	11,89	8,73	9,41	5,91	5,76	18,03	16,01	14,41	13,3	4,89	15,34	8,58	6,7	8,02	4,81	6,3	6,11	9,78	8,11	17,94	11,4	8,36	12,91
		Apical	4,16	9,94	13,11	13,09	8,62	5,65	17,47	18,03	10,83	9,95	4,78	7,35	6,83	9,29	8,14	7,19	3,23	3,59	13,18	18,24	5,36	14,77	8,55	12,85
		Mean	4,08	8,8	10,22	11,05	7,41	5,32	16,32	15,69	11,44	11,07	4,91	10,08	11,81	11,35	7,11	5,63	6,77	5,21	10,04	9,94	12,41	14,69	7,89	10,94
